# Flat-panel Detector Perfusion Imaging and Conventional Multidetector Perfusion Imaging in Patients with Acute Ischemic Stroke: A Comparative Study

**DOI:** 10.1007/s00062-024-01401-7

**Published:** 2024-03-25

**Authors:** Bettina L. Serrallach, Adnan Mujanovic, Nikolaos Ntoulias, Michael Manhart, Mattia Branca, Alex Brehm, Marios-Nikos Psychogios, Christoph C. Kurmann, Eike I. Piechowiak, Sara Pilgram-Pastor, Thomas Meinel, David Seiffge, Pasquale Mordasini, Jan Gralla, Tomas Dobrocky, Johannes Kaesmacher

**Affiliations:** 1 Department of Diagnostic and Interventional Neuroradiology, Inselspital, Bern University Hospital, University of Bern, Freiburgstrasse, 3010 Bern, Switzerland; 2 Department of Neuroradiology, Clinic for Radiology and Nuclear Medicine, University Hospital Basel, Petersgraben 4/Spitalstrasse 21, 4031 Basel, Switzerland; 3 Advanced Therapies, Siemens Healthcare GmbH, Siemensstrasse 1, 91301 Forchheim, Germany; 4 CTU Bern, University of Bern, Mittelstrasse 43, 3012 Bern, Switzerland; 5 Department of Neurology, Inselspital, Bern University Hospital, University of Bern, Freiburgstrasse, 3010 Bern, Switzerland; 6 Department of Radiology, Netzwerk Radiologie, Kantonsspital St. Gallen, Rorschacher Strasse 95, 9007 St. Gallen, Switzerland; 7 Graduate School for Health Sciences, University of Bern, Bern, Switzerland; 8 Department of Diagnostic, Interventional and Pediatric Radiology, Inselspital, Bern University Hospital, University of Bern, Freiburgstrasse, 3010 Bern, Switzerland

**Keywords:** Infarct core, Penumbra, Time to peak, Time to maximum, Relative cerebral blood flow

## Abstract

**Purpose:**

Flat-panel detector computed tomography (FDCT) is increasingly used in (neuro)interventional angiography suites. This study aimed to compare FDCT perfusion (FDCTP) with conventional multidetector computed tomography perfusion (MDCTP) in patients with acute ischemic stroke.

**Methods:**

In this study, 19 patients with large vessel occlusion in the anterior circulation who had undergone mechanical thrombectomy, baseline MDCTP and pre-interventional FDCTP were included. Hypoperfused tissue volumes were manually segmented on time to maximum (Tmax) and time to peak (TTP) maps based on the maximum visible extent. Absolute and relative thresholds were applied to the maximum visible extent on Tmax and relative cerebral blood flow (rCBF) maps to delineate penumbra volumes and volumes with a high likelihood of irreversible infarcted tissue (“core”). Standard comparative metrics were used to evaluate the performance of FDCTP.

**Results:**

Strong correlations and robust agreement were found between manually segmented volumes on MDCTP and FDCTP Tmax maps (r = 0.85, 95% CI 0.65–0.94, *p* < 0.001; ICC = 0.85, 95% CI 0.69–0.94) and TTP maps (r = 0.91, 95% CI 0.78–0.97, *p* < 0.001; ICC = 0.90, 95% CI 0.78–0.96); however, direct quantitative comparisons using thresholding showed lower correlations and weaker agreement (MDCTP versus FDCTP Tmax 6 s: r = 0.35, 95% CI −0.13–0.69, *p* = 0.15; ICC = 0.32, 95% CI 0.07–0.75). Normalization techniques improved results for Tmax maps (r = 0.78, 95% CI 0.50–0.91, *p* < 0.001; ICC = 0.77, 95% CI 0.55–0.91). Bland-Altman analyses indicated a slight systematic underestimation of FDCTP Tmax maximum visible extent volumes and slight overestimation of FDCTP TTP maximum visible extent volumes compared to MDCTP.

**Conclusion:**

FDCTP and MDCTP provide qualitatively comparable volumetric results on Tmax and TTP maps; however, direct quantitative measurements of infarct core and hypoperfused tissue volumes showed lower correlations and agreement.

**Supplementary Information:**

The online version of this article (10.1007/s00062-024-01401-7) contains supplementary material, which is available to authorized users.

## Introduction

In recent decades, flat-panel detector technology has become integral to (neuro)interventional angiography suites [1–5]. Applications of flat-panel detector computed tomography (FDCT) include initial diagnostic imaging in the one-stop management workflow [6, 7], assessment of acute periprocedural complications in the angiography suite [3, 4] and detection of residual occlusions and their anatomic location during and shortly after neurovascular procedures. Recently introduced multiphase FDCT perfusion (FDCTP) acquisition protocols are capable of providing time-resolved whole brain dynamic perfusion imaging, including cerebral blood flow (CBF) and time to maximum (Tmax) maps [5, 8]. In acute ischemic stroke (AIS), perfusion imaging aids in the detection of occluded vessels (especially in peripheral occlusions) and influences therapeutic decision making [9–11]. Perfusion maps help to visualize the volume of critically hypoperfused, irreversibly lost tissue regardless of subsequent reperfusion status (“infarct core”) and the hypoperfused, potentially salvageable ischemic penumbra (“tissue at risk”) [11–15].

Unlike conventional multidetector computed tomography (MDCT), FDCT can be acquired directly in the angiography suite. Thus, it provides additional and potentially crucial information for pre-interventional and peri-interventional decision making [1, 2, 16] and increases the likelihood of improved clinical outcomes [2, 16]. The MDCT may be considered the reference standard but given the increasing availability and use of FDCT, it is essential to establish comparability between imaging results obtained with the two techniques. The differences in acquisition techniques, which are primarily related to temporal resolution, such as volume acquisition time and time between consecutive acquisitions should be taken into account [5, 17]. This comparability is crucial to provide a reliable basis for optimal therapeutic decisions.

However, to date, studies evaluating the comparability of new-generation FDCTP and conventional MDCTP are still scarce [5, 17, 18]. Therefore, the aim of this study was to assess the comparability of new-generation FDCTP and conventional MDCTP.

## Methods

This study adhered to the principles outlined in the Declaration of Helsinki and received approval by the local ethics committee. The requirement for active informed patient consent was waived due to the retrospective nature of this study.

Consecutive patients with AIS, treated between June 2019 and March 2021 were selected from the neuroradiologic database. Patients who fulfilled the following additional criteria were included in this study: (a) they had undergone mechanical thrombectomy for anterior circulation stroke, (b) received a baseline conventional MDCTP and a pre-interventional FDCTP of diagnostic quality in the angiography suite, (c) had no change of occlusion between baseline CT angiography and the first digital subtraction angiography run (e.g., M1 occlusion on both images) and (d) a time interval < 120 min between the conventional MDCTP and the FDCTP. Of the 19 patients identified, 16 were “mothership” patients, and 3 “drip and ship” patients. Clinical data were extracted from the institutional stroke database or from the clinical information system.

### Workflow

Following clinical evaluation by a neurologist in the emergency department, patients underwent conventional stroke protocol imaging, which included both computed tomography angiography (CTA) and computed tomography perfusion (CTP). If the eligibility criteria for mechanical thrombectomy were met, patients were transferred to the angiography suite and pre-interventional FDCT imaging was acquired at the treating physician’s discretion. The decision to perform FDCT imaging was based on clinical judgment and the aim was to provide additional information to assist the physicians in modifying the treatment strategy, where appropriate.

### Image Acquisition

#### MDCT

The MDCT data were acquired using conventional multislice CT scanners (Somatom Definition Edge, Siemens Healthineers, Somatom Definition AS+, Siemens Healthineers, Erlangen, Germany; Revolution, GE Healthcare, Chicago, IL, USA). The CTP imaging was performed according to a standard protocol as recommended by the manufacturer. A dual-head power injector was used to deliver 30 ml of the contrast agent (Iopamiro 400, Bracco, Switzerland) at a rate of 5 ml/s through an 18‑G venous line. This was followed by a 30 ml saline flush. The acquisition parameters used for most of the images were as follows: 350 mA, 80 kV, 570 ms, a matrix size of 512 × 512, a field of view of 20 cm, a spiral pitch factor of 0.5, a single collimation width of 1.2 mm, and H20f kernel. A total of 30 contrast phases were acquired in 46.3–50.0 s. The acquisition parameters for all MDCTP images can be found in the Supplementary Table 1.

#### FDCT

Full-brain FDCT imaging data were acquired using a biplane flat-panel detector angiographic system (ARTIS Icono, Siemens Healthineers) [5, 19, 20]. For FDCTP, a total of 10 rotational sweeps of the angiographic C‑arm system with a duration of 5 s each and a 1‑s turnaround were performed. The first two rotations served as mask runs, and the subsequent eight rotations documented the inflow and outflow of contrast agent (60 ml of Iopamiro 400, Bracco), yielding concentration measurements of contrast agent at eight time points. An 18‑G venous line for contrast injection and the subsequent 40–60 ml saline flush (mono-head or dual-head power injector at an injection rate of 5 ml/s) was used. The injection is started together with the start of the acquisition of the first rotational sweep. A filtered back projection-based algorithm, available on the clinical system was used to reconstruct each acquired rotation individually (0.48 mm voxel size, 512 × 512 matrix, 378 slices, 0.48 mm slice thickness, and reconstruction kernel “HU Normal”) [5, 19, 20].

### Image Postprocessing

#### MDCT Perfusion Postprocessing

The MDCTP data were analyzed semi-automatically using standard perfusion postprocessing software (syngo.via, Siemens Healthineers) on a dedicated workstation [20, 21]. Standard algorithms were adapted as follows: HU thresholds of −100–200 HU, smoothing strength of 12 mm, slice thickness of 3 mm and by disabling the vessel suppression.

#### FDCT Perfusion Postprocessing

An offline prototype software package (Siemens Healthineers) was used to compute the FDCT perfusion maps [20]. Compensation for potential head motion during acquisition was achieved by a 3D-3D registration between the first acquired volume and all remaining volumes. The volumes were resampled to 1 mm voxel size and automatically aligned to the orbitomeatal line. The first two mask volumes served as a template to enable the anatomical background to be subtracted from the remaining eight contrasted volumes. Nonlinear filtering was applied to the contrasted subtracted volumes to reduce noise [22], and resampling of the subtracted volume series via temporal spline interpolation generated the time-concentration curves (temporal sampling of 1 s). The arterial input function was detected automatically and voxels containing air, bone or vascular structures were excluded by thresholding. Deconvolution-based perfusion analysis was used to calculate perfusion maps [23]. All computed perfusion maps were stored in a 16-bit raster data format for visualization in the 3D Slicer software, version 5.2.1 [24]. Using the 3D Slicer software, the FDCTP data were reformatted to 3 mm slice thickness.

### Volume Segmentation

The maximum visible extent of the hypoperfused tissue on Tmax and TTP maps was manually delineated on a slice per slice basis using 3D Slicer [24]. The MDCTP and FDCTP studies were assessed independently by a board-certified radiologist and a neuroradiology fellow, blinded to clinical presentation and to all other imaging studies performed. The readers were instructed to perform their assessment and report the volumes (in ml) on a standardized spreadsheet, and a short educational module was provided before beginning the image interpretation. The area of maximum visible extent on Tmax maps was subjected to a threshold (MDCTP and FDCTP: > 6 s), and the corresponding volumes were calculated. To generate relative cerebral blood flow (rCBF) maps, CBF maps were normalized to the contralateral semioval center as reference tissue by using the average value obtained from 3 regions of interest each 7mm in size. The regions of interest were positioned in the anterior, middle, and posterior thirds of the semioval center, respectively. The following thresholds were used: < 30% for MDCTP and < 30% as well as < 45% for FDCTP [5, 11]. To reduce false-positive low CBF regions, the thresholds were applied to the voxels within the manual segmentation of Tmax.

In the next stage, a semi-automated volume computation was performed (offline DynaCT perfusion prototype for ARTIS Icono systems) using the MDCTP and FDCTP Tmax and CBF maps, based on normalized Tmax and CBF maps. For normalization, mean Tmax and CBF values of the brain tissue in both hemispheres were computed and the hemisphere not affected by the index stroke was identified by the lower mean Tmax and the higher CBF value. A normalization factor was found by computing the median value of all Tmax or CBF values within the tissue in the healthy hemisphere. The normalized Tmax and CBF maps were computed by dividing all Tmax or CBF values by the normalization factor and multiplying by 100 to calculate relative percentage values. Voxels with increased Tmax values were segmented by thresholding the normalized Tmax maps above 150% and restricting the segmented area to the prior manual segmentation. Voxels with decreased CBF values were segmented by thresholding the normalized CBF maps below 30% and restricting the segmented area to the prior manual segmentation. The infarct volume was computed from the volume of all segmented voxels.

### Statistical Analysis

Results are presented as median with interquartile range (IQR) and frequencies with percentages (%), unless otherwise noted. Continuous variables were evaluated using Wilcoxon-Mann-Whitney U‑tests. The correlation of baseline MDCTP and FDCTP volumes, stratified by the time between the scans, is displayed in scatter plots. In addition, Pearson’s correlation coefficient (r), the coefficient of determination (r^2^) and the intraclass coefficient (ICC) were calculated. Bland-Altman analysis was performed to compare the two imaging techniques. The overall agreement between the raters and the different imaging techniques was calculated using a mixed effect model with random intercept varying among the imaging techniques and the raters within the imaging methods. The agreement between the imaging techniques only was also calculated using a mixed-effect model with random intercept varying among the patients. The estimates are shown with their 95% confidence intervals (CI). All statistical analyses were performed with R (version 4.3.0) [25].

## Results

Of 32 patients for whom pre-interventional standard cross-sectional perfusion imaging and pre-interventional FDCTP imaging were available, 8 were excluded because they underwent magnetic resonance perfusion imaging as baseline imaging and 5 because of insufficient imaging quality, leaving a final cohort of 19 patients (Supplementary Fig. 1). Relevant baseline characteristics are shown in Table 1.

**Table 1:** Patient baseline characteristics and hypoperfusion volumes (in ml) on multidetector computed tomography perfusion (MDCTP) and flat-panel detector computed tomography perfusion (FDCTP)

**Included patients (*n*)**	19
**Baseline characteristics**	
Female *n*, (%)	8 (42)
Age (years), median (IQR)	79 (62, 83)
Time between scans (min), median (IQR)	47 (42, 71)
Baseline NIHSS, median (IQR)	14 (11, 19)
Occluded vessel (%)	
M1	13 (68.4)
M2	6 (31.6)
Intravenous thrombolysis *n*, (%)	7 (36.8)
**Volumes (ml)**	
MDCTP Tmax (visible), median (IQR)	221.9 (163.9, 255.2)
MDCTP Tmax (> 6s), median (IQR)	102.6 (60.8, 135.2)
FDCTP Tmax (visible), median (IQR)	214.3 (178.2, 249.0)
FDCTP Tmax (> 6s), median (IQR)	99.7 (24.8, 133.7)
MDCTP infarct core (rCBF < 30%), median (IQR)	17.4 (13.7, 29.6)
FDCTP infarct core (rCBF < 30%), median (IQR)	15.3 (3.6, 26.0)
FDCTP infarct core (rCBF < 45%), median (IQR)	34.8 (10.5, 50.5)
MDCTP mismatch (Tmax visible-rCBF < 30%), median (IQR)	191.0 (146.1, 224.0)
MDCTP mismatch (Tmax>6 s-rCBF < 30%), median (IQR)	62.3 (38.9, 105.6)
FDCTP mismatch (Tmax visible-rCBF < 30%), median (IQR)	181.5 (154.4, 227.3)
FDCTP mismatch (Tmax visible-rCBF < 45%), median (IQR)	162.6 (132.5, 202.7)
FDCTP mismatch (Tmax>6 s-rCBF < 30%), median (IQR)	66.6 (16.0, 115.5)
FDCTP mismatch (Tmax>6 s-rCBF < 45%), median (IQR)	51.2 (0.5, 100.0)
MDCTP TTP (visible), median (IQR)	226.8 (173.9, 268.2)
FDCTP TTP (visible), median (IQR)	247.1 (171.2, 280.7)
MDCTP Tmax (normalized), median (IQR)	187.1 (140.3, 222.6)
FDCTP Tmax (normalized), median (IQR)	190.5 (140.6, 227.1)
MDCTP rCBF (normalized), median (IQR)	8.0 (4.6, 15.2)
FDCTP rCBF (normalized), median (IQR)	5.0 (0.10, 10.30)

*FDCTP* flat-panel detector computed tomography perfusion, *IQR* interquartile range, *MDCTP* multidetector computed tomography perfusion, *NIHSS* National Institutes of Health Stroke Scale, *rCBF* relative cerebral blood flow, *Tmax* time to maximum, *TTP* time to peak

The overall agreement for the volumes between the raters and different imaging techniques was assessed using a mixed effects model. The calculated agreement ranged from 0.88 (95% CI 0.80–0.95) to 1.0 (95% CI 0.99–1.0), indicating reliable ratings.

**Fig. 1:**
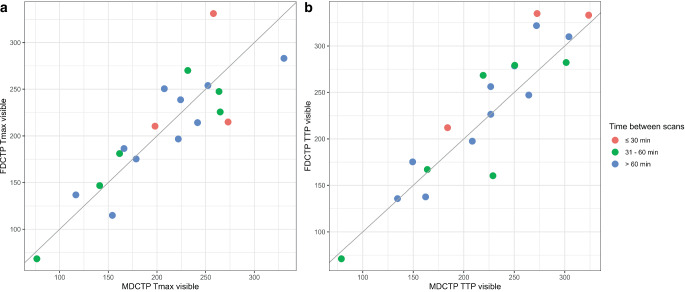
Scatterplots depicting the relationship between the volumes (in ml) of the segmented maximum visible extent of hypoperfused tissue on flat-panel detector computed tomography perfusion (FDCTP) and on multidetector computed tomography perfusion (MDCTP), stratified by times between the scans. **a** There is a strong and positive linear relationship between the volumes of the maximum visible extent on MDCTP time to maximum (MDCTP Tmax visible) and on FDCTP Tmax (FDCTP Tmax visible). **b** Similarly, there is a strong positive linear relationship between the volumes of the maximum visible extent on MDCTP time to peak (MDCTP TTP visible) and FDCTP TTP (FDCTP TTP visible)

**Fig. 2:**
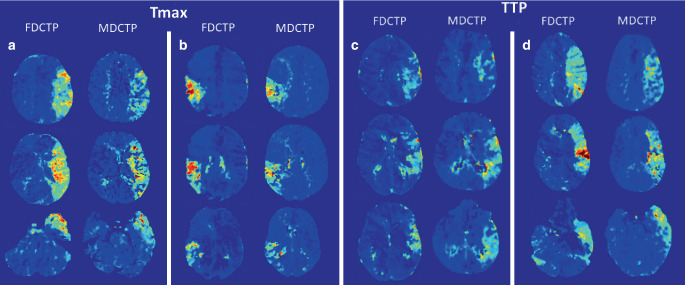
Examples of corresponding hypoperfusion patterns on time to maximum (Tmax) (**a**, **b**) and time to peak (TTP) maps (**c**, **d**) obtained from flat-panel detector computed tomography perfusion (FDCTP) and multidetector computed tomography perfusion (MDCTP). Each column represents three exemplary axial images of the corresponding hypoperfused tissue. Jet color coding from blue to red was used, with red colors indicating delayed perfusion compared to non-hypoperfused areas coded in dark blue. Comparable extensions can be found on FDCTP and MDCTP Tmax maps for the left M1 occlusion (**a**) and the right M2 occlusion (**b**), as well as on TTP maps from FDCTP and MDCTP for the two examples of left M1 occlusion (**c**, **d**)

A strong correlation and robust agreement was observed between the volume of hypoperfused tissue as determined by the manually segmented maximum visible extent on MDCTP Tmax and FDCTP Tmax (r = 0.85, 95% CI 0.65–0.94, *p* < 0.001; ICC = 0.85, 95% CI 0.69–0.94; Figs. 1 and 2**, **Table 2). Similar results were obtained when comparing the volume of hypoperfused tissue on MDCTP TTP with FDCTP TTP (r = 0.91, 95% CI 0.78–0.97, *p* < 0.001; ICC = 0.90, 95% CI 0.78–0.96; Figs. 1 and 2**, **Table 2). Furthermore, in both cases, a significant proportion of the variance was shared by the two variables (MDCTP visible Tmax versus FDCTP visible Tmax: r^2^ = 0.73; MDCTP visible TTP versus FDCTP visible TTP: r^2^ = 0.83). The comparison between the results of the other variables showed lower correlations, weaker agreements, and less shared variance (Table 2).

**Table 2:** Comparisons between multidetector computed tomography perfusion (MDCTP) and flat-panel detector computed tomography perfusion (FDCTP)

Multidetector computed tomography perfusion (MDCTP) versus flat-panel detector computed tomography perfusion (FDCTP)	Pearson’s correlation coefficient (r), intraclass coefficient (ICC), difference in contrast between the methods (DC) and coefficient of determination (r^2^)
MDCTP Tmax visible versus FDCTP Tmax visible	r = 0.85, 95% CI 0.65–0.94, *p* < 0.001; ICC = 0.85, 95% CI 0.69–0.94; DC = −0.82 95%, CI −15.65–14.01, *p* = 0.914; r^2^ = 0.73
MDCTP TTP visible versus FDCTP TTP visible	r = 0.91, 95% CI 0.78–0.97, *p* < 0.001; ICC = 0.90, 95% CI 0.78–0.96; DC = 9.11, 95% CI −4.52–22.74, *p* = 0.19; r^2^ = 0.83
MDCTP Tmax 6 s versus FDCTP Tmax 6 s	r = 0.35, 95% CI −0.13–0.69, *p* = 0.15; ICC = 0.32, 95% CI 0.07–0.75; DC = −5.81, 95% CI −37.81–26.18, *p* = 0.72; r^2^ = 0.12
MDCTP infarct core rCBF < 30% versus FDCTP infarct core rCBF < 30%	r = 0.38, 95% CI −0.09–0.71, *p* = 0.11; ICC = 0.37, 95% CI 0.10–0.76; DC = −5.60, 95% CI −21.68–10.49, *p* = 0.50; r^2^ = 0.15
MDCTP infarct core rCBF < 30% versus FDCTP infarct core rCBF < 45%	r = 0.30, 95% CI −0.180.66, *p* = 0.22; ICC = 0.29, 95% CI 0.05–0.75; DC = 14.68, 95% CI −5.39–34.75, *p* = 0.15; r^2^ = 0.09
MDCTP mismatch (maximal visible extent—rCBF < 30%) versus FDCTP mismatch (maximal visible extent—rCBF < 30%)	r = 0.73, 95% CI 0.41–0.89, *p* < 0.001; ICC = 0.72, 95% CI 0.47–0.88; DC = 4.78, 95% CI −14.09–23.65, *p* = 0.62; r^2^ = 0.53
MDCTP mismatch (maximal visible extent—rCBF < 30%) versus FDCTP mismatch (maximal visible extent—rCBF < 45%)	r = 0.65, 95% CI 0.28–0.85, *p* = 0.003; ICC = 0.63, 95% CI 0.35–0.85; DC = −15.50, 95% CI −36.11–5.11, *p* = 0.14; r^2^ = 0.42
MDCTP mismatch (Tmax > 6 s—rCBF < 30%) versus FDCTP mismatch (Tmax 6 s—rCBF < 30%)	r = 0.29, 95% CI −0.19–0.65, *p* = 0.24; ICC = 0.27, 95% CI 0.04–0.75; DC = −0.22, 95% CI −30.12–29.69, *p* = 0.99; r^2^ = 0.08
MDCTP mismatch (Tmax > 6 s—rCBF < 30%) versus FDCTP mismatch (Tmax 6 s—rCBF < 45%)	r = 0.19, 95% CI −0.29–0.60, *p* = 0.43; ICC = 0.19, 95% CI 0.01–0.80; DC = −20.49, 95% CI −49.25–8.26, *p* = 0.16; r^2^ = 0.04
MDCTP Tmax 6 s versus FDCTP semi-automated normalized Tmax	r = 0.73, 95% CI 0.41–0.89, *p* < 0.001; ICC = 0.69, 95% CI 0.43–0.87; DC = 82.86, 95% CI 62.73–102.98, *p* < 0.001; r^2^ = 0.53
MDCTP semi-automated normalized Tmax versus FDCTP semi-automated normalized Tmax	r = 0.78, 95% CI 0.50–0.91, *p* < 0.001; ICC = 0.77, 95% CI 0.55–0.91; DC = 1.45, 95% CI −17.75–20.64, *p* = 0.88; r^2^ = 0.60
MDCTP semi-automated normalized rCBF versus FDCTP semi-automated normalized rCBF	r = 0.16, 95% CI −0.32–0.57, *p* = 0.57; ICC = 0.16, 95% CI 0.01–0.84; DC = −3.82, 95% CI −14.46–6.81, *p* = 0.48; r^2^ =0.03

*rCBF* relative cerebral blood flow, *Tmax* time to maximum, *TTP* time to peak

In general, the variations in perfusion volumes between MDCTP and FDCTP remained consistent regardless of the time interval between scans (Tmax: r = 0.02, 95% CI −0.44–0.47, *p* = 0.93; TTP: r = 0.28, 95% CI −0.20–0.65, *p* = 0.25; Supplementary Fig. 2) or patient age (Tmax: r = 0.18, 95% CI −0.29–0.59, *p* = 0.45; TTP: r = 0.3, 95% CI −0.17–0.67, *p* = 0.21).

Of the 19 patients 7 received intravenous thrombolysis (36.8%). For the Tmax maps, the variation in perfusion volumes determined by the manually segmented maximum visible extent on MDCTP and FDCTP did not differ between patients who received and those who did not receive intravenous thrombolysis (difference MDCTP-FDCTP: median 3.3 ml, IQR −12.9–23.8 ml, and median −7.1 ml, IQR −24.2–25.7 ml for with and without intravenous alteplase, *p* = 0.71). For TTP maps, a difference was observed between patients who received intravenous thrombolysis and those who did not (difference MDCTP-FDCTP: median: 8.0 ml, IQR −3.7–21.9 ml, and median −27.0 ml, IQR −34.4–−2.3 ml for with and without intravenous alteplase, *p* = 0.03); however, these volume differences determined by the manually segmented maximum visible extent between MDCTP and FDCTP, remained consistent regardless of the time interval between the initiation of intravenous thrombolysis and the start of the FDCTP (Tmax: r = −0.17, 95% CI −0.82–0.67, *p* = 0.72; TTP: r = 0.66, 95% CI −0.19–0.94, *p* = 0.11).

Bland-Altman analysis for visible Tmax showed a bias of 0.82 ml (95% CI −15.5–17.2 ml) and a range of agreement levels from −65.6–67.2 ml (Supplementary Fig. 3a). Visible MDCTP TTP volumes were lower compared with FDCTP TTP (mean bias −9.11 ml, 95% CI −24.1–5.9 ml; agreement levels −70.1–51.9 ml) (Supplementary Fig. 3b).

The comparison between the volume of hypoperfused tissue as determined by MDCTP Tmax 6 s (not normalized) and the semi-automated computed normalized Tmax maps of FDCTP yielded a strong correlation and good agreement (r = 0.73, 95% CI 0.41–0.89, *p* < 0.001; ICC = 0.69, 95% CI 0.43–0.87). A significant proportion of the variance in the volume of hypoperfused tissue is shared by MDCTP and FDCTP (MDCTP Tmax 6 s versus FDCTP semi-automated normalized Tmax: r^2^ = 0.53).

**Fig. 3:**
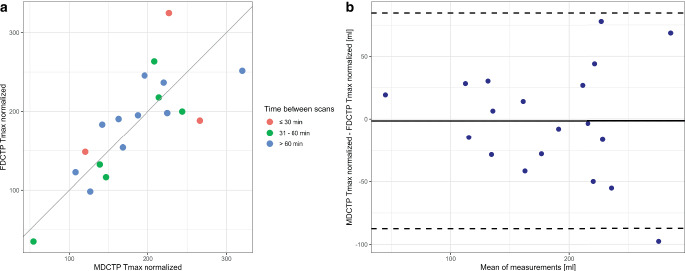
**a** Scatterplot illustrating the correlation between the volumes (in ml) of hypoperfused tissue on the semi-automated normalized time to maximum (Tmax) maps of flat-panel detector computed tomography perfusion (FDCTP Tmax normalized) and on multidetector computed tomography perfusion (MDCTP Tmax normalized), stratified by times between the scans. There is a strong and positive linear relationship. **b** Bland-Altman plot displaying the differences in volumes obtained on MDCTP and FDCTP for the semi-automated normalized Tmax. The mean difference (bias, solid line) and 95% CI of agreement (dashed lines) are shown

The comparison between semi-automated computed normalized Tmax maps of MDCTP and FDCTP yielded improved results (in comparison to MDCTP Tmax 6 s not normalized and the semi-automated computed normalized Tmax maps of FDCTP) with a strong correlation and robust agreement between the volume of hypoperfused tissue (r = 0.78, 95% CI 0.50–0.94, *p* < 0.001; ICC = 0.77, 95% CI 0.55–0.91; Fig. 3). Furthermore, a significant proportion of the variance was shared by the two variables (MDCTP semi-automated normalized Tmax versus FDCTP semi-automated normalized Tmax: r^2^ = 0.60).

The comparison between semi-automated computed normalized rCBF maps of MDCTP and FDCTP showed low correlation and poor agreement, and minimal shared variance of volumes of infarct core (r = 0.16, 95% CI −0.32–0.57, *p* = 0.57; ICC = 0.16, 95% CI 0.01–0.84; r^2^ = 0.03).

Bland-Altman analysis for the semi-automated normalized Tmax (MDCTP and FDCTP) showed a bias of −1.45 ml (95% CI −22.6–19.7 ml) and a range of agreement levels from −87.4 to 84.5 ml.

## Discussion

This study shows that FDCTP and MDCTP provide similar volumetric results when qualitatively assessing hypoperfused tissue on Tmax and TTP maps; however, direct quantitative comparison between infarct core and hypoperfused tissue on FDCTP and MDCTP using thresholding did not yield satisfactory correlations or agreements. For Tmax maps, normalization techniques proved effective in addressing this issue and led to improved results. A possible reason for the limited quantitative comparability between FDCTP and MDCTP is the inherent differences in acquisition techniques. These differences primarily concern temporal resolution, such as the time required to acquire a volume (approximately 0.5 s for MDCT versus 5 s for FDCT), the time between two consecutive acquisitions (approximately 2 s for MDCT versus 6 s for FDCT), and the number of sweeps (30 for MDCTP versus 10 for FPCTP).

On average, the qualitatively calculated volumes (segmented maximum visible extent on Tmax maps) were almost equal for MDCTP and FDCTP (0.82 ml, 95% CI −15.5–17.2 ml). By contrast, the overall volumes of tissue determined on the semi-automated normalized Tmax were smaller with MDCTP than with FDCTP (−1.45 ml, 95% CI −22.6–19.7 ml). This pattern was also observed for TTP maps (−9.11 ml, 95% CI −24.1–5.9 ml). The increased bias observed in TTP maps compared with Tmax maps may be due to measurement errors or possibly, although not significant, some degree of greater sensitivity of TTP maps to time, as volumes tend to expand with increasing duration of vessel occlusion. Because TTP maps are calculated before deconvolution, they are more sensitive to variations such as cardiac function, proximal vessel stenosis, and bolus injection quality [15].

In an exploratory analysis, smaller volumes of hypoperfusion were found on FDCTP in patients pretreated with intravenous thrombolysis, despite no substantial change in occlusion location when comparing MDCTP and FDCTP. This difference was statistically significant on TTP maps. While we cannot exclude that prior administration of intravenous thrombolysis may have had an effect on collateral flow or microcirculatory changes leading to differences in hypoperfused volume, this may also be a chance finding and the analysis is limited by the fact that only 7 of 19 patients received intravenous thrombolysis with alteplase.

Whereas our study found that quantitative results from MDCTP and FDCTP were comparable only for Tmax and not for CBF maps after normalization, previous studies have used the RAPID software (RAPID for ANGIO, iSchemaView) to quantitatively compare hypoperfused areas on MDCTP using rCBF < 30% with FDCTP using rCBF < 45%, as well as MDCTP Tmax > 6 s with FDCTP Tmax > 6 s [5, 26]. These studies demonstrated a strong correlation between these two modalities [5, 26]. In clinical practice, most institutions still rely primarily on qualitative assessment of maps rather than measuring absolute values in different regions of the affected tissue. In addition, existing literature suggests that even in the presence of limited quantitative differences in the infarct core, penumbra, and mismatch estimates between different software packages, their influence on radiologists’ decisions regarding endovascular treatment remains relatively small [27]; however, given the potential benefits it would be advantageous to develop reliable thresholds and automated approaches for comparing MDCTP and FDCTP. Up to now, accepted thresholds for MDCTP identifying infarct core and/or hypoperfused regions include rCBF < 30% and Tmax > 6 s [5, 11, 15], while proposed thresholds for FDCTP involve rCBF < 45% and Tmax > 6 s [5, 26]; however, several studies have reported an overestimation of perfusion deficits on FDCTP [28, 29]. Moreover, Zussman et al. showed that notable discrepancies can occur in quantitative MDCTP results obtained with different vendor software applications, primarily due to vendor differences rather than interoperator or intraoperator variability [30].

The acquired protocol reported here offers the possibility to grade collaterals on multiphase CTA (mCTA) images, which has been shown previously [19]. While multiphase or even single-phase CTA could be considered sufficient to select patients for thrombectomy, evaluating the reliability of a “full” perfusion imaging protocol for FPCTP is important because perfusion imaging after thrombectomy can provide important information, including an assessment of the eloquence of residual small occlusions and can help to assess whether tissue distal to residual vessel occlusions is still viable.

Numerous studies have conclusively demonstrated the practicality of FDCTP in the angiography suite [5, 8, 28, 29, 31, 32]. Petroulia et al. evaluated the comparability of FDCT and MDCT and found equal diagnostic performance in the supratentorial ventricular system and high specificity and sensitivity for the detection of intracranial hemorrhages, with the best diagnostic performance in detecting intraventricular hemorrhage, followed by intraparenchymal and subarachnoid hemorrhages [33]. High sensitivity and specificity were also demonstrated in the detection of ischemic lesions [33]; however, they found limitations in the diagnostic performance between MDCT and FDCT in all infratentorial structures and in the grey-white matter differentiation of the supratentorial and infratentorial structures [33]. Hoelter et al. compared FDCT to MDCT, including a comparison of FDCTP to MDCTP, and found that FDCTP datasets had equivalent image quality to MDCTP, providing the information needed to estimate infarct core and penumbra [34]. Another study by Niu et al. found that after applying postprocessing methods to enhance image quality, FDCTP maps were not inferior to MDCTP maps [35]. In addition, radiation exposure during FDCTP is comparable to that during MDCTP, with estimated effective doses of 4.52 mSv (without collimation) and 2.88 mSv (with collimation) [36] and both utilized similar volumes of contrast agent [28]. FDCTP is still primarily used in the one-stop management workflow [2]. If proven to provide reliable, reproducible and comparable results to MDCTP, this could pave the way towards wider implementation of FDCTP in clinical practice. The use of FDCTP imaging in the peri-interventional and post-interventional settings of AIS, for instance, has the potential to provide significant benefits to patients. Currently, the management of patients with incomplete reperfusion is challenging due to the scarcity of robust evidence to support treatment decisions [37]; however, if FDCTP performed peri-interventionally and post-interventionally is shown to provide additional reliable and relevant information, it could have a profound impact on the clinical management of these patients. Gaining proficiency in using the FDCT as a conventional CT scanner and interpreting its imaging results will be critical for translating research findings into improved patient care [5].

The distinct advantages of MRI over CT in detecting the infarct core, lacunar infarcts, and posterior circulation infarcts, are due to the high sensitivity and specificity of diffusion-weighted imaging [11]. This has led to the increased use of MRI as a baseline imaging modality. In addition, a comparison of FDCTP with post-interventional magnetic resonance perfusion (MRP) would be useful. There is a growing need to compare MRP with CTP by establishing new thresholds. Campbell et al. demonstrated that quantitative mismatch classification using rCBF and Tmax in MDCTP is similar to the MRI perfusion-diffusion mismatch [38]; however, MRI perfusion is inherently less quantitative than CTP. Also, direct comparison of perfusion parameters between the two modalities is difficult due to the nonproportional relationship between signal and contrast agent concentration, as well as technical and mathematical differences [11, 17].

This study has several limitations. First, the results are based on a retrospective study. Second, the sample size is relatively small. Third, the study included only patients with large vessel occlusion of the anterior circulation. Fourth, the FDCTP was performed by a single neurointerventionalist. Finally, we only compared MDCTP with FDCTP and did not include an evaluation of MRP. To address these limitations, future studies should aim for larger sample sizes and include patients with more distal occlusions, patients with posterior circulation occlusions, and patients with baseline MRI perfusion to gather more robust evidence and increase the generalizability of these findings. In addition, consideration of “smart” acquisition protocols tailored to contrast boluses has the potential to address inherent differences between MDCTP and FDCTP and merits future exploration.

## Conclusion

This study showed that flat-panel detector computed tomography perfusion (FDCTP) provides robust volumetric results when qualitatively assessing hypoperfusion on time to maximum (Tmax) and time to peak (TTP) maps, with a high degree of agreement and strong correlation with the results of MDCTP; however, direct quantitative comparisons using thresholding methods did not provide satisfactory correlations. Nevertheless, for Tmax maps the application of normalization techniques proved effective in overcoming this limitation and led to improved results. Further research is needed to investigate the comparability of FDCTP with MDCTP more thoroughly. If proven reliable and consistent, the use of FDCTP in clinical practice could be greatly expanded, opening new avenues for improved patient care.

### Supplementary Information

Supplementary Fig. 1: Patients flowchart
Supplementary Table 1: Acquisition parameters used for MDCTP
Supplementary Fig. 2: Differences of volumes for MDCTP and FDCTP in correlation with the corresponding time between the scans
Supplementary Fig. 3: Bland-Altman plots showing the differences in volumes of maximum visible extent obtained on MDCTP and FDCTP for Tmax and TTP

## Abbreviations

AIS: Acute ischemic stroke
CBF: Cerebral blood flow
CI: Confidence interval
CTA: Computed tomography angiography
CTP: Computed tomography perfusion
FDCTP: Flat-panel detector computed tomography perfusion
ICC: Intraclass coefficient
IQR: Interquartile range
MDCTP: Multidetector computed tomography perfusion
MRP: Magnetic resonance perfusion
r: Pearson’s correlation coefficient
r^2^: Coefficient of determination
rCBF: Relative cerebral blood flow
Tmax: Time to maximum
TTP: Time to peak
