# Correction to: Flat-panel Detector Perfusion Imaging and Conventional Multidetector Perfusion Imaging in Patients with Acute Ischemic Stroke

**DOI:** 10.1007/s00062-024-01410-6

**Published:** 2024-06-04

**Authors:** Bettina L. Serrallach, Adnan Mujanovic, Nikolaos Ntoulias, Michael Manhart, Mattia Branca, Alex Brehm, Marios-Nikos Psychogios, Christoph C. Kurmann, Eike I. Piechowiak, Sara Pilgram-Pastor, Thomas Meinel, David Seiffge, Pasquale Mordasini, Jan Gralla, Tomas Dobrocky, Johannes Kaesmacher

**Affiliations:** 1 Department of Diagnostic and Interventional Neuroradiology, Inselspital, Bern University Hospital, University of Bern, Freiburgstrasse, 3010 Bern, Switzerland; 2 Department of Neuroradiology, Clinic for Radiology and Nuclear Medicine, University Hospital Basel, Petersgraben 4/Spitalstrasse 21, 4031 Basel, Switzerland; 3 Advanced Therapies, Siemens Healthcare GmbH, Siemensstrasse 1, 91301 Forchheim, Germany; 4 CTU Bern, University of Bern, Mittelstrasse 43, 3012 Bern, Switzerland; 5 Department of Neurology, Inselspital, Bern University Hospital, University of Bern, Freiburgstrasse, 3010 Bern, Switzerland; 6 Department of Radiology, Netzwerk Radiologie, Kantonsspital St. Gallen, Rorschacher Strasse 95, 9007 St. Gallen, Switzerland; 7 Graduate School for Health Sciences, University of Bern, Bern, Switzerland; 8 Department of Diagnostic, Interventional and Pediatric Radiology, Inselspital, Bern University Hospital, University of Bern, Freiburgstrasse, 3010 Bern, Switzerland

**Correction to:**

**Clin Neuroradiol 2024**

10.1007/s00062-024-01401-7

In this article the affiliation details for Author Alex Brehm were incorrectly given as ‘Advanced Therapies, Siemens Healthcare GmbH, Siemensstrasse 1, 91301 Forchheim, Germany’ but should have been ‘Department of Neuroradiology, Clinic for Radiology and Nuclear Medicine, University Hospital Basel, Petersgraben 4/Spitalstrasse 21, 4031 Basel, Switzerland’.

The affiliation details for Author Marios-Nikos Psychogios were incorrectly given as ‘Advanced Therapies, Siemens Healthcare GmbH, Siemensstrasse 1, 91301 Forchheim, Germany’ but should have been ‘Department of Neuroradiology, Clinic for Radiology and Nuclear Medicine, University Hospital Basel, Petersgraben 4/Spitalstrasse 21, 4031 Basel, Switzerland’.

The affiliation details for Author Pasquale Mordasini were incorrectly given as ‘Department of Radiology, Netzwerk Radiologie, Kantosspital St Gallen, Rorschacher Straße 95, 9007 St. Gallen, Switzerland’ but should have been ‘Department of Radiology, Netzwerk Radiologie, Kantonsspital St. Gallen, Rorschacher Strasse 95, 9007 St. Gallen, Switzerland’.

The original article has been corrected.

