# Supplementary material for: Flat-panel Detector Perfusion Imaging and Conventional Multidetector Perfusion Imaging in Patients with Acute Ischemic Stroke: A Comparative Study
Source: Clin Neuroradiol. 2024 Mar 25;34(3):625–35. doi: 10.1007/s00062-024-01401-7 (PMC11339100; doi:10.1007/s00062-024-01401-7)
Supplement: Supplementary file 1 — Supplementary Fig. 1: Patients flowchart Supplementary Table 1: Acquisition parameters used for MDCTP Supplementary Fig. 2: Differences of volumes for MDCTP and FDCTP in correlation with the corresponding time between the scans Supplementary Fig. 3: Bland-Altman plots showing the differences in volumes of maximum visible extent obtained on MDCTP and FDCTP for Tmax and TTP [file 62_2024_1401_MOESM1_ESM.docx]

**Supplementary Information**


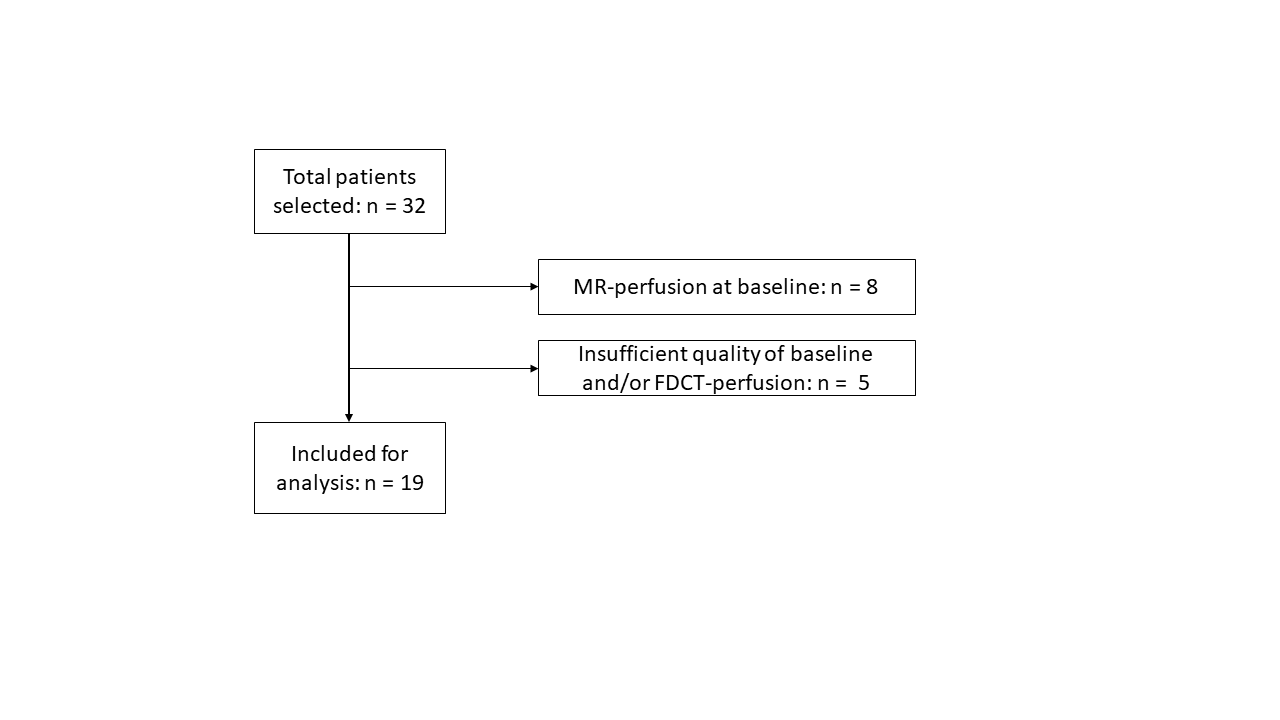


**Supplementary Fig. 1** Flowchart depicting the patient selection

**Supplementary Table 1** Acquisition parameters used for multidetector computed tomography perfusion

| **Patients (n)** | **Acquisition parameters** |
| --- | --- |
| 14 | 350 mA, 80 kV, 570 ms, a matrix size of 512 × 512, a field of view of 20 cm, a spiral pitch factor of 0.5, a single collimation width of 1.2 mm, and H20f kernel. A total of 30 contrast phases were acquired. |
| 2 | 180 mA, 70 kV, 500 ms, a matrix size of 512 × 512, a field of view of 20 cm, a spiral pitch factor of 0.5, a single collimation width of 1.2 mm, and Hr36f kernel. A total of 28 contrast phases were acquired. |
| 1 | 273 mA, 80 kV, 660 ms, a matrix size of 512 × 512, a field of view of 20 cm, a spiral pitch factor of 0.5, a single collimation width of 1.2 mm, and H20f kernel. A total of 29 contrast phases were acquired. |
| 1 | 304 mA, 80 kV, 660 ms, a matrix size of 512 × 512, a field of view of 20 cm, a spiral pitch factor of 0.5, a single collimation width of 1.2 mm, and H20f kernel. A total of 30 contrast phases were acquired. |
| 1 | 120 mA, 80 kV, 350 ms, a matrix size of 512 × 512, a field of view of 22 cm, axial acquisition, a single collimation width of 0.625 mm, and standard kernel. A total of 25 contrast phases were acquired. |


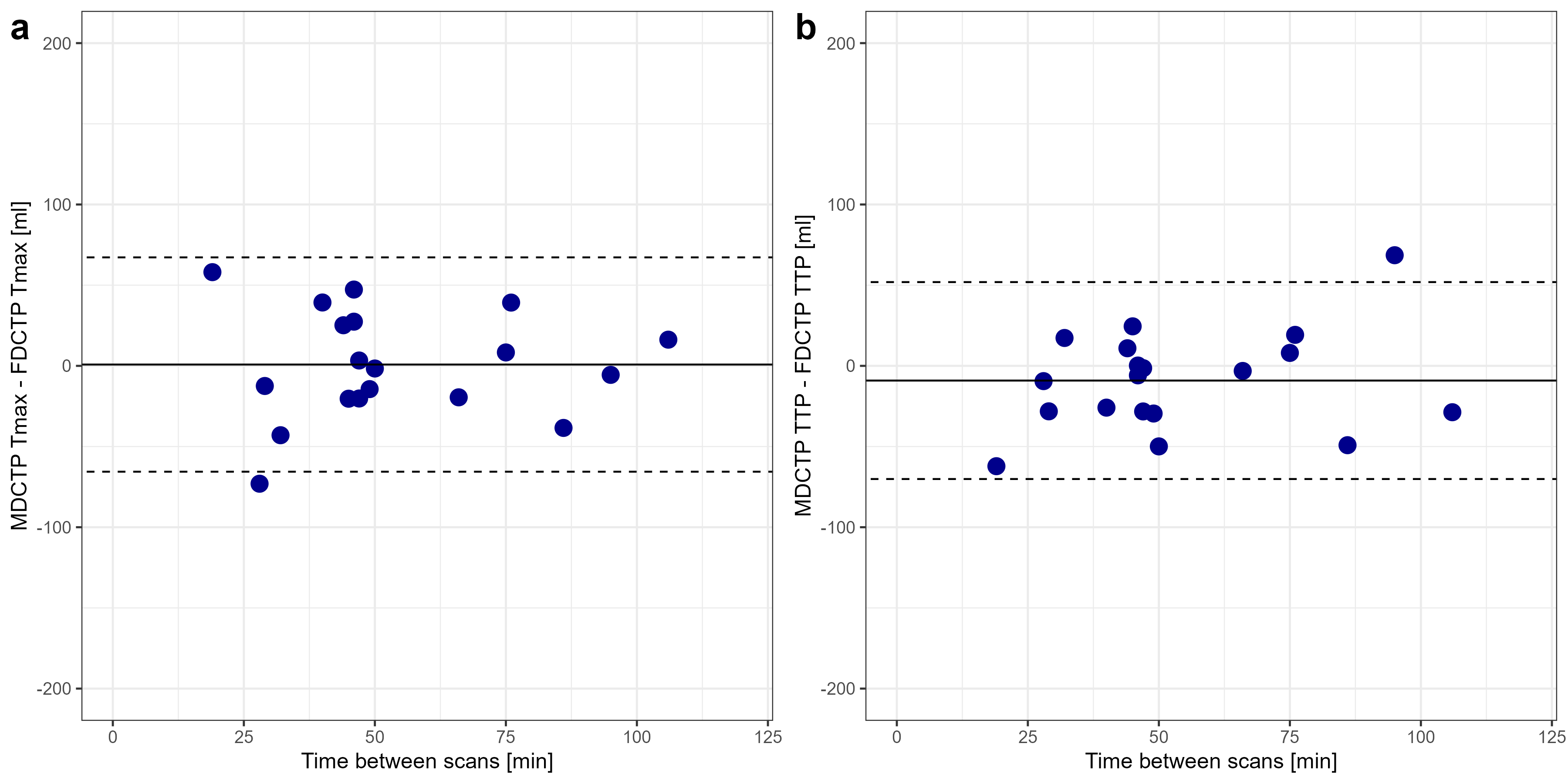


**Supplementary Fig. 2** Differences of volumes for multidetector computed tomography perfusion (MDCTP) and flat-panel detector computed tomography perfusion (FDCTP) in correlation with the corresponding time between the scans. (**a**) For time to maximum (Tmax), and (**b**) for time to peak (TTP). Mean of differences (solid line) and limits of agreement are shown (dashed lines).


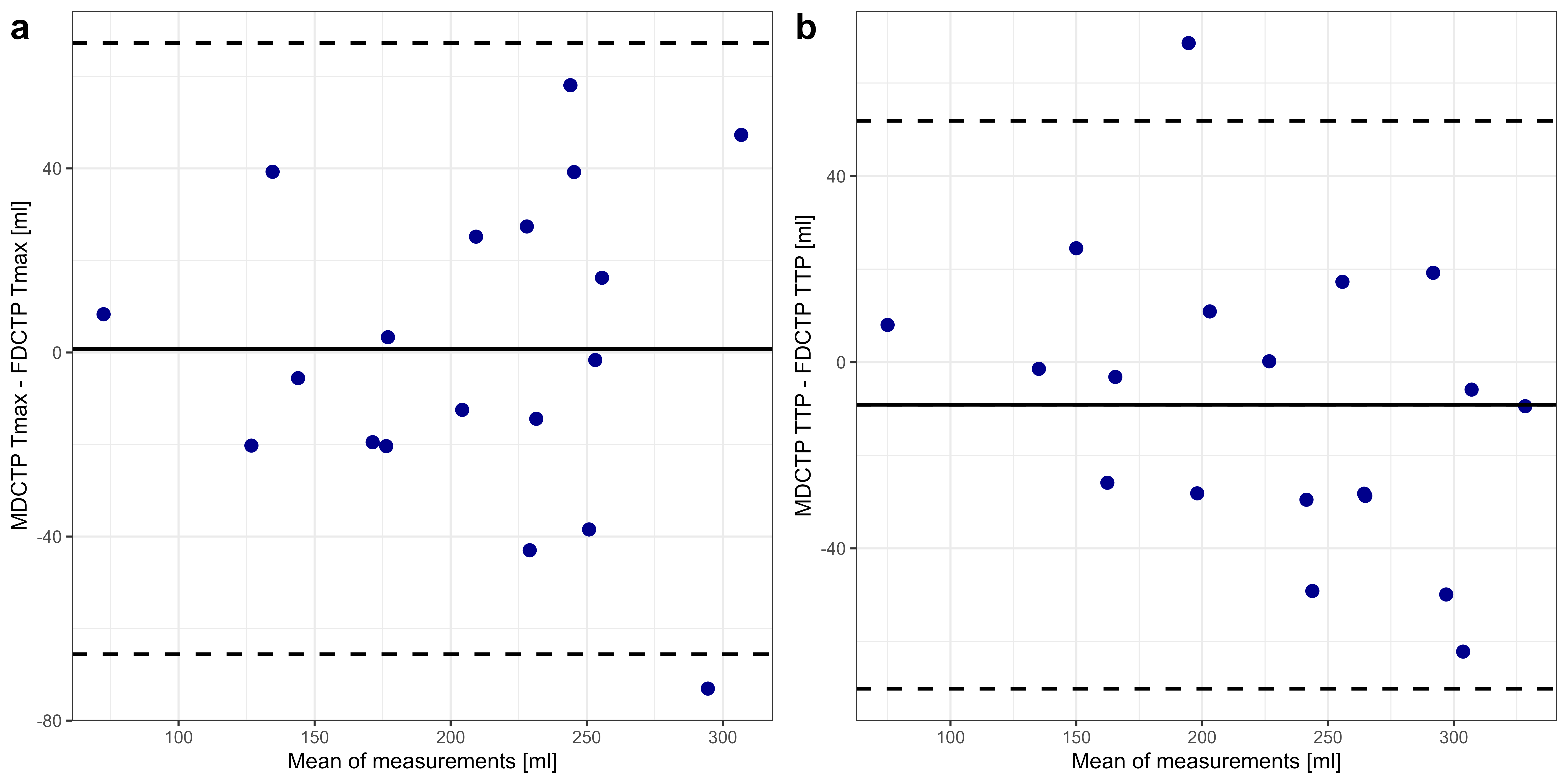


**Supplementary Fig. 3** Bland-Altman plots showing the differences in volumes of maximum visible extent obtained on multidetector computed tomography perfusion (MDCTP) and flat-panel detector computed tomography perfusion (FDCTP) for (**a**) time to maximum (Tmax) and (**b**) time to peak (TTP). The mean difference (bias, solid line) and 95% CI of agreement (dashed lines) are shown.
